# Knockdown of circular RNA septin 9 inhibits the malignant progression of breast cancer by reducing the expression of solute carrier family 1 member 5 in a microRNA-149-5p-dependent manner

**DOI:** 10.1080/21655979.2021.2000731

**Published:** 2021-12-11

**Authors:** Jianjun Wang, Kunxian Yang, Junyu Cao, Li Li

**Affiliations:** Department of Breast and Thyroid Tumors Surgery, The First People’s Hospital of Yunnan Province, Kunhua Hospital Affiliated to Kunming University of Science and Technology, Yunnan, China

**Keywords:** Breast cancer, circSEPT9, miR-149-5p, SLC1A5

## Abstract

Breast cancer (BC) is the most frequently diagnosed cancer in women. Increasing evidence suggests that circular RNA (circRNA) exerts critical functions in BC progression. However, the roles of circRNA septin 9 (circSEPT9) in BC development and the underneath mechanism remain largely unclear so far. In this work, the RNA levels of circSEPT9, microRNA-149-5p (miR-149-5p) and solute carrier family 1 member 5 (SLC1A5) were detected by quantitative real-time polymerase chain reaction. Western blot was performed to check protein expression. Glutamine uptake, cell proliferation and cell apoptosis were investigated by glutamine uptake, cell counting kit-8, cell colony formation, 5-Ethynyl-29-deoxyuridine, flow cytometry analysis or DNA content quantitation assay. The interactions of miR-149-5p with circSEPT9 and SLC1A5 were identified by a dual-luciferase reporter assay. Mouse model assay was carried out to analyze the effect of circSEPT9 on tumor formation *in vivo*. Results showed that circSEPT9 and SLC1A5 expression were significantly upregulated, while miR-149-5p was downregulated in BC tissues and cells as compared with paracancerous normal breast tissues and human normal breast cells. Knockdown of circSEPT9 or SLC1A5 inhibited glutamine uptake and cell proliferation, but induced cell apoptosis in BC cells. SLC1A5 overexpression relieved circSEPT9 silencing-induced repression of BC cell malignancy. In mechanism, circSEPT9 regulated SLC1A5 expression by sponging miR-149-5p. In support, circSEPT9 knockdown led to delayed tumor tumorigenesis *in vivo*. In summary, these results indicates that circSEPT9 may act an oncogenic role in BC malignant progression by regulating miR-149-5p/SLC1A5 pathway, providing a novel mechanism responsible for BC development.

## Introduction

Accounting for almost 15% of cancer-caused deaths, breast cancer (BC) is the most frequent malignancy for women [1]. As evaluated, there were more than 2 million new BC patients and 626,679 breast cancer deaths in 2018 globally [2]. Adjuvant therapy and hormonal therapy improve the clinical outcome of BC patients, but display severe side effects [3]. As a result, long-term survival rate remains urgent clinical problems. Thus, the sustained efforts to reveal the key regulators related to BC development are of great necessity for the increase of recovery rate.

Circular RNA (circRNA) is an endogenous noncoding transcript that generally forms a single-stranded covalently circular structure by back-splicing or skipping event [4]. CircRNA has a variety of vital biological roles like regulator of gene transcription, sponge of microRNA (miRNA), protein scaffold, recruiter of specific protein and sponge of RNA binding protein [5]. Increasing evidence has suggested the key roles of circRNA in carcinogenesis and cancer progression [6]. Besides, multiple circRNAs are dysregulated in BC progression and involved in the biological processes related to BC development, such as circ_001783 [7], circ_0003645 [8] and circ_103809 [9]. However, there are few data about the role of circRNA septin 9 (circSEPT9) in BC malignant progression.

Disorders of cellular metabolism are regarded as hallmarks of cancer [10]. Glutamine is a versatile amino acid that is a basic metabolic resource involved in energy formation and regulatory pathways in cancer cells [11]. As reported, the involvement of glutamine in aberrant bioenergetics promotes energy formation, survival, and growth, and inhibits production of thioredoxin-interacting protein, which negatively regulates glucose consumption [12]. Mounting studies indicate that glutamine metabolism is a potential target for the therapy of cancers [13]. Solute carrier family 1 member 5 (SLC1A5) belongs to the member of solute carrier 1 and is a glutamine transporter [14]. SLC1A5 is commonly highly expressed in cancer and can inhibit the biological behaviors of cancer cells by reducing glutamine uptake [15]. In particular, previous data have indicated that SLC1A5 participates in breast cancer progression through regulation of glutamine uptake [16].

MiRNA is highly conserved noncoding RNA that can post-transcriptionally modulate gene expression through promoting their degradation or restraining their translation [17]. Abnormal expression of miRNA has been considered as a culprit of BC progression. For example, miR-146 introduction promoted BC cell growth through interaction with NME/NM23 nucleoside diphosphate kinase1 (NM23-H1) *in vitro* [18]. MiR-16-5p downregulation enhanced the proliferation and migration of BC cells by increasing AKT serine/threonine kinase3 (AKT3) [19]. Another miRNA, miR-149-5p, commonly acts a tumor-repressing role, and its specific role has been reported in gastric cancer (GC) [20], colorectal cancer [21], ovarian cancer [22] and breast cancer [23].

Coincidently, as predicted by bioinformatics tools, circSEPT9 and 3ʹ-untranslated region (3ʹUTR) of SLC1A5 carried the binding sites of miR-149-5p. Based on the above contents, we hypothesize that circSEPT9 participates in the progression of BC through miR-149-5p/SLC1A5 pathway. However, these is no data about the mechanism of circSEPT9 in BC progression. Thus, the study is designed to determine whether BC progression involves circSEPT9/miR-149-5p/SLC1A5 pathway, so as to provide therapeutic targets for BC.

## Materials and methods

### Clinical BC specimens

Under the approval from the Ethics Committee of The First People’s Hospital of Yunnan Province, sixty BC patients from The First People’s Hospital of Yunnan Province were recruited for the collection of BC tissues and matched healthy breast tissues. All participants provided the written informed consent before curative resection. The collected samples were kept at −80°C in a freezer.

### Cell lines

Human normal breast cells (MCF-10A), BC cell lines (MDA-MB-231 and BT-549) and human embryonic kidney cells (293 T) were purchased from Procell (Wuhan, China). MCF-10A, BT-549 and 293 T cells were cultured in Dulbecco’s modified Eagle’s medium (DMEM)/F12 (Gibco, Carlsbad, CA, USA), Roswell Park Memorial Institute-1640 (RPMI-1640; Gibco) and DMEM (Gibco), respectively, at 37°C in an incubator with 5% CO_2_. MDA-MB-231 cells were grown in Leibovitz’s L15 (Thermo Fisher, Waltham, MA, USA) at 37°C in an atmosphere of 100% air. The addition of serum, antibiotics, epidermal growth factor and insulin was performed according to the standard protocols.

### Quantitative real-time polymerase chain reaction (qRT-PCR) and identification of circRNA stability

Actinomycin D treatment assay was executed using Actinomycin D (Rechemscience, Shang, China) for 0, 8, 12 and 24 h. TransZol (TransGen, Beijing, China) was used to isolate RNA from BC tissues and cells. RNA quality was measured using NanoDrop-1000 apparatus (Thermo Fisher). A part of isolated RNA was employed for the incubation with RNase R (4 U/μg RNA; Geneseed, Guangzhou, China) in a 37°C incubator for 20 min. Reverse transcription of RNA was carried out using high-capacity cDNA synthesis kits (Thermo Fisher). SYBR Green Super Mix (Roche, Basel, Switzerland) was utilized to determine gene expression as instructed. RNA expression was normalized to U6 or β-actin by the 2^−∆∆Ct^ method. The primer sequences are presented in Table 1.

**Table 1.** Primers sequences used for qRT-PCR

Name		Sequences (5ʹ-3ʹ)
circSEPT9 (hsa_circ_0005320)	Forward	CAGCCCAGCCCAGACCTT
	Reverse	CGACCTCCTCGACCTCAAAA
SEPT9	Forward	AAGAAGTCTTACTCAGGAGGCA
	Reverse	GGAGTTGGGTGTCTCGACCT
miR-149-5p	Forward	GGCTCTGGCTCCGTGTCTT
	Reverse	CAGTGCAGGGTCCGAGGTATT
SLC1A5	Forward Reverse	CCACCCTCCCGGACCTAA GGTCTGCAGGAGGCTAGGTT
β-actin	Forward	CACCATTGGCAATGAGCGGTTC
	Reverse	AGGTCTTTGCGGATGTCCACGT
U6	Forward	CTTCGGCAGCACATATACT
	Reverse	AAAATATGGAACGCTTCACG

### Cell transfection

The small hairpin RNAs against circSEPT9 (sh-circSEPT9, 5ʹ-GCCAGGAGGCCTTGAAAAGAT-3ʹ) and SLC1A5 (sh-SLC1A5, 5ʹ-TCAGCAGCCTTTCGCTCATACTCTA-3ʹ), miR-149-5p mimic (5ʹ-UCUGGCUCCGUGUCUUCACUCCC-3ʹ), miR-149-5p inhibitor (5ʹ-GGGAGUGAAGACACGGAGCCAGA-3ʹ) and respective controls (sh-NC, sh-con, mimic NC and anti-miR-NC) were synthesized by Ribobio Co., Ltd. (Guangzhou, China). pcDNA 3.1 vector (pcDNA-NC) and full-length SLC1A5 were used to generate SLC1A5 overexpression plasmid (pcDNA-SLC1A5). Cell transfection was executed using FuGENE6 (Roche) following the user’s manual.

### Glutamine uptake assay

The assay involving the detection of glutamine uptake was performed as shown previously [24]. In brief, the cells were digested using trypsin (Thermo Fisher) and suspended in glutamine-free media containing ^3^H-labeled glutamine (Perkin Elmer, Boston, MA, USA) lasting 5 min. Next, the cells were spun down and lysed using SDS/0.2 N natrium hydroxydatum solution. After neutralized with HCl, these samples were analyzed with a scintillation counter (Perkin Elmer).

### Cell proliferation

For cell counting kit-8 (CCK-8) assay, MDA-MB-231 and BT-549 cells were premixed with media and added into 96-well microplates (4000 cells per well). After treated with sh-circSEPT9, sh-NC, GPNA (1 mM), dimethyl sulfoxide (DMSO, control), sh-SLC1A5, sh-con, pcDNA-SLC1A5 and pcDNA-NC, the cells were cultured for 0, 24, 48 and 72 h. After that, CCK-8 kit (Beyotime, Shanghai, China) was used to analyze these cells as instructed. Finally, all samples (at a wavelength of 450 nm) were detected using a microplate reader (Thermo Fisher).

Cell colony formation assay was performed as shown previously [25], 500 cells were placed in each well of 6-well petri dishes, and cultured for 14 days under the standard culture conditions. Next, the cells were fixed and stained using paraformaldehyde (Phygene, Fuzhou, China) and crystal violet (Phygene), respectively. Cell proliferation was confirmed by assessing the number of positive colonies containing at least 50 cells.

For 5-Ethynyl-29-deoxyuridine (EDU) assay, BC cells were seeded in 6-well petri dishes and treated with test compounds for 48 h. Then, EDU-labeled medium was added into 96-well plates, and cells were cultured for 2 h, followed by analyzing using EDU labelling/detection kit (Ribobio) according to the standard protocol [26]. Results were determined by analyzing the percentage of EDU-positive cells from 5 random fields.

For DNA content quantitation assay, the cells with various treatments were harvested by centrifugation. After their concentrations were adjusted to 1 × 10^6^/mL cells, the cells were fixed using ethanol (Millipore, Bradford, MA, USA). The cells were subjected to incubation with RNase A (Solarbio, Beijing, China) and propidium iodide (PI). At last, samples were analyzed using a flow cytometer (Thermo Fisher).

### Cell apoptosis analysis

With respect to cell apoptosis, Annexin V-FITC apoptosis detection kit (Solarbio) was executed following the instruction of manufacture. In brief, BC cells were collected and diluted in Binding buffer. A mixture containing Annexin V-FITC and PI was used to incubate the cells. Finally, cell apoptosis was determined with a flow cytometer (Thermo Fisher).

### Western blot analysis

Tissue and cell lysates were prepared according to the standard instructions [27] after the indicated treatments. After clearing by centrifugation, equivalent amounts of protein lysates, as determined by protein concentration determination kit (Solarbio), were loaded onto SurePAGE gels, blotted onto nitrocellulose membranes and incubated with primary antibodies against proliferating cell nuclear antigen (PCNA), BCL2-associated x protein (Bax), B-cell lymphoma-2 (Bcl-2), SLC1A5 and β-actin. Finally, the signals were visualized by RapidStep ECL Reagent (Millipore). All antibodies were purchased from Thermo Fisher Scientific and used in a dilution of 1:1000. β-actin acted as a control gene reference.

### Dual-luciferase reporter assay

The detailed methods were reported previously [28]. In brief, the binding sites of miR-149-5p with circSEPT9 and SLC1A5 were predicted through circbank (http://www.circbank.cn/index.html) or starbase (http://starbase.sysu.edu.cn/agoClipRNA.php?source=mRNA) online database, and the wild-type (wt) and mutant (mut) circSEPT9 were synthesized by Geneseed Co., Ltd., which were later inserted into the pmirGLO plasmid (Miaoling, Wuhan, China) using T4 DNA Ligase (Roche). Similarly, the wild-type and mutant plasmids of SLC1A5 were generated, and termed as SLC1A5 3ʹUTR wt and SLC1A5 3ʹUTR mut. Then, the transfection of miR-149-5p mimic, mimic NC and the above reporter plasmids was performed on the indicated BC cells using FuGENE6 (Roche). The binding intensity was confirmed with a Dual-Lucy Assay Kit (Solarbio) following a 48-hour incubation.

### Mouse model assay

Five-week-old BALB/C nude mice (N = 12) were bought from Charles River (Beijing, China), and fed in pathogen-free cabinets. Five million of MDA-MB-231 cells stably expressing sh-circSEPT9 or sh-NC were injected into separate sides of mice back. The volume of the primary tumors from MDA-MB-231 cells was measured every 1 week for 4 cycles based on the formula that length × width^2^ × 0.5. At the 28th day, all mice were euthanatized using xylazine (Seebio Biotech, Shanghai, China), followed by the collection of neoplasms. The harvested peritoneal nodules were stored in ultralow temperature freezer for further analysis of tumor weight and gene expression. The study was permitted by the Animal Care and Use Committee of The First People’s Hospital of Yunnan Province.

### Immunohistochemistry (IHC) for nuclear proliferation marker (ki-67) and PCNA

As instructed [29], the neoplasms harvested in mouse model assay were cut into 4-μm thickness prior to embeddedness into paraffin. The sections were heated at 80°C to perform antigen retrieval. After blocking endogenous peroxidase activity with H_2_O_2,_ the proteins were probed with the primary antibodies specific to ki-67 (1:1000; Thermo Fisher) and PCNA (1:500; Thermo Fisher). The subsequent steps were executed using the IHC assay kit (Phygene) as per guidebook. Eventually, the slides were covered with coverslips for the capture of staining results.

### Statistical analysis

The experimental statistics analysis was performed on GraphPad Prism and image J software. Student’s *t*-tests or Wilcoxon rank-sum test was involved in the comparison between the two groups, while analysis of variance was implicated in the disparities among three or more groups. Data were expressed as means ± standard deviations. The statistical significance was considered when *P* value <0.05.

## Results

The function of circSEPT9 in BC development remains largely unclear so far. According to bioinformatics predictions, we hypothesizes that circSEPT9 participates in the progression of BC through miR-149-5p/SLC1A5 pathway. The goal of the present study is to reveal the specific role of circSEPT9 in BC development and the inner mechanism. Our results showed that circSEPT9 was overexpressed in BC tissues and cells, and its knockdown inhibited BC cell proliferation and glutamine uptake but induced cell apoptosis. Further, we found that circSEPT9/miR-149-5p/SLC1A5 pathway was responsible for BC development.

### CircSEPT9 expression was significantly increased in BC tissues and cells

**Figure 1.**
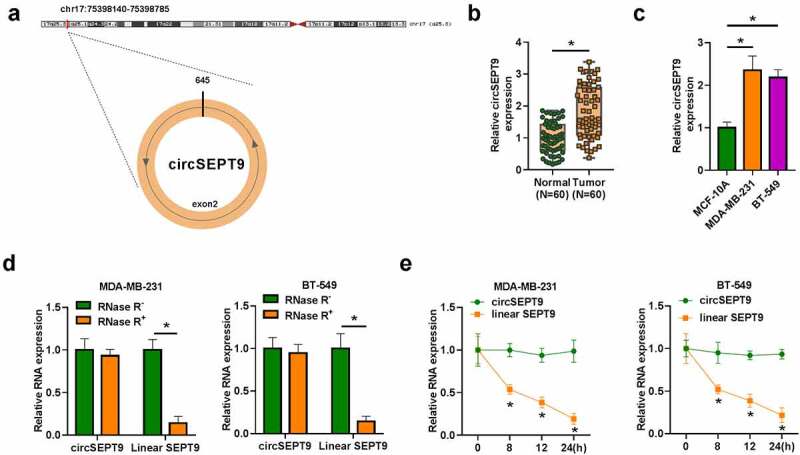
**Expression of circSEPT9 in BC tissues and cells**. (a) The production of circSEPT9. (b and c) qRT-PCR was used to detect circSEPT9 expression in 60 pairs of BC and paracancerous healthy breast tissues, and MCF-10A, MDA-MB-231 and BT-549 cells. (d and e) The stability of circSEPT9 was confirmed by RNase R and Actinomycin D treatment assays. *P < 0.05

CircSEPT9 was located in chr17:75,398,140–75,398,785 and formed by exon 2 of SEPT9 gene (Figure 1a). The data from Figure 1b and c showed that circSEPT9 was overexpressed in BC tissues and cell lines (MDA-MB-231 and BT-549) in comparison with adjacent normal breast tissues or human normal breast cell-line MCF-10A. Meanwhile, we found that circSEPT9 expression was significantly associated with tumor size, distant metastasis, tumor-node-metastasis (TNM) stage and HER-2 status (Table S1). Besides, the study analyzed the stability of circSEPT9 in both MDA-MB-231 and BT-549 cells. As shown in Figure 1d, RNase R treatment significantly reduced the expression of linear SEPT9, but not circSEPT9 expression. Moreover, it was found using Actinomycin D that circSEPT9 had a longer half-life than linear SEPT9 (Figure 1e). Collectively, the above data demonstrates that circSEPT9 may be involved in BC progression.

### CircSEPT9 knockdown inhibited glutamine uptake and cell proliferation, but induced cell apoptosis in MDA-MB-231 and BT-549 cells

**Figure 2.**
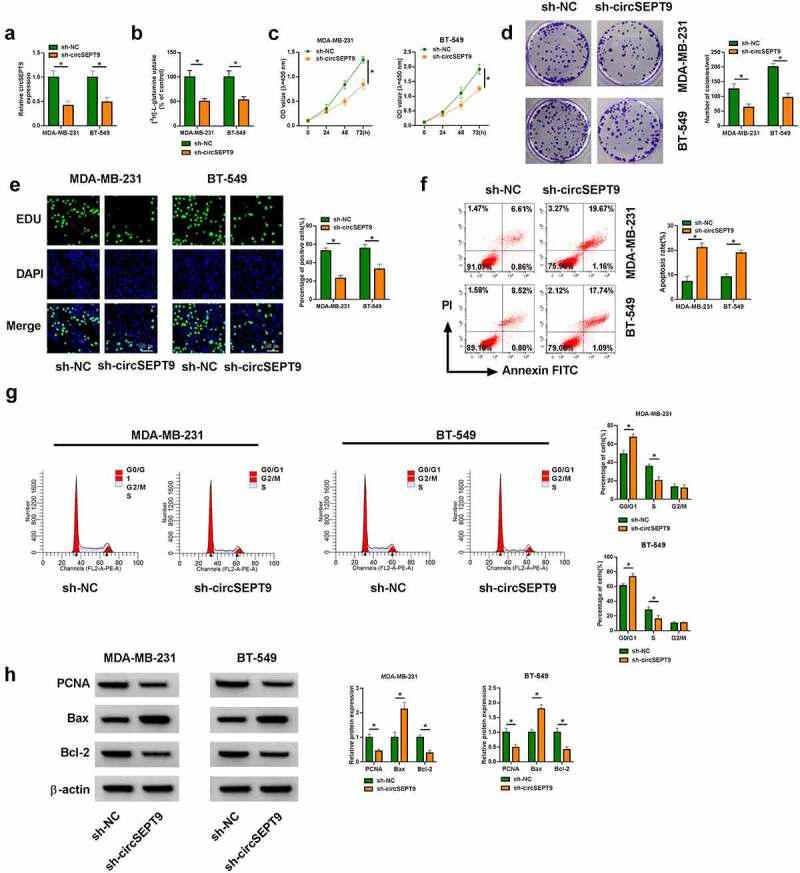
**The effects of circSEPT9 knockdown on glutamine uptake, cell proliferation and cell apoptosis in MDA-MB-231 and BT-549 cells**. (a-h) Both MDA-MB-231 and BT-549 cells were transfected with sh-NC and sh-circSEPT9, respectively, and circSEPT9 expression was determined by qRT-PCR (a), glutamine uptake by glutamine uptake assay (b), cell proliferation by CCK-8, cell colony formation, EDU and DNA content quantitation assays (C, D, E and G), cell apoptosis by flow cytometry (f), and the protein expression of PCNA, Bax and Bcl-2 by Western blot (h). *P < 0.05

Whether circSEPT9 participated in BC progression was first analyzed *in vitro*. As shown in Figure 2a, the small hairpin RNA targeting circSEPT9 (sh-circSEPT9) was effective in decreasing circSEPT9 expression. Subsequently, circSEPT9 silencing inhibited glutamine uptake (Figure 2b). The optical density (OD) values and the number of positive colonies and EDU-positive cells were reduced after circSEPT9 knockdown (Figure 2c-E). Consistently, circSEPT9 deficiency induced cell apoptosis and cell cycle arrest at G0/G1 phase (figure 2f and G). In support, Western blot analysis testified the inhibitory effects of circSEPT9 depletion on BC cell malignancy. For instance, reduced expression of circSEPT9 led to decreases of PCNA and Bcl-2 protein expression and an increase of Bax expression (Figure 2h). Thus, these findings demonstrates that circSEPT9 silencing represses the malignant behaviors of BC cells.

### SLC1A5 inhibitors inhibited glutamine uptake and cell proliferation, but promoted cell apoptosis in MDA-MB-231 and BT-549 cells

**Figure 3.**
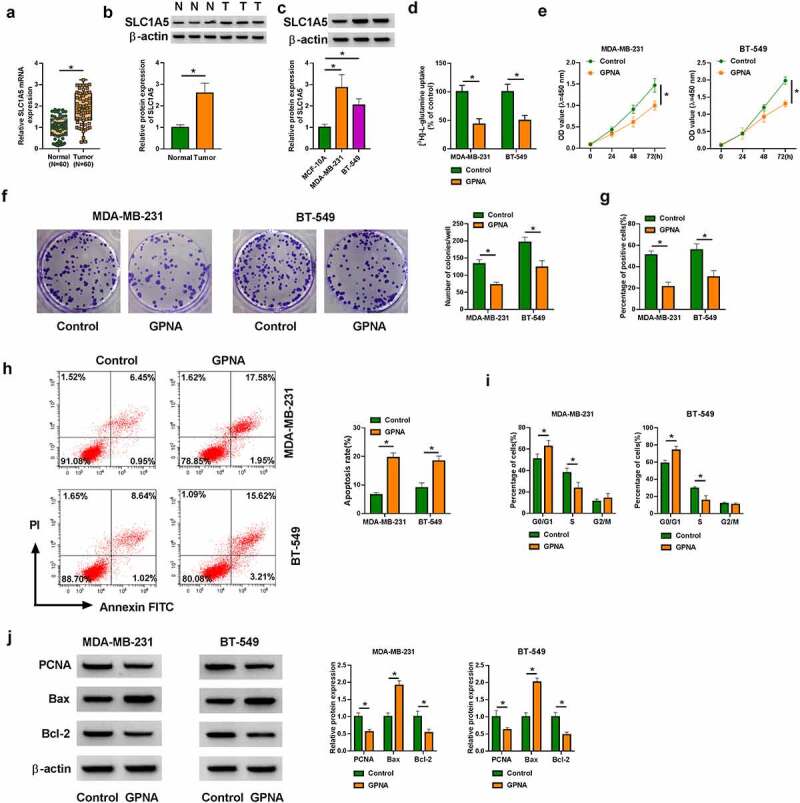
**GPNA treatment inhibited MDA-MB-231 and BT-549 cell processes**. (a-c) SLC1A5 expression at mRNA and protein level was detected by qRT-PCR and Western blot analysis, respectively, in BC tissues, paracancerous healthy breast tissues, MCF-10A cells, MDA-MB-231 cells and BT-549 cells. (d-j) Both MDA-MB-231 and BT-549 cells were treated with 1 mM GPNA, with the cells treated with DMSO acted as a control, and glutamine uptake was analyzed by glutamine uptake assay (d), cell proliferation by CCK-8, cell colony formation, EDU and DNA content quantitation assays (E, F, G and I), cell apoptosis by flow cytometry analysis (h), and the protein expression of PCNA, Bax and Bcl-2 by Western blot (j). *P < 0.05

Then, we continued to explore the effects of SLC1A5 on BC cell processes. To validate this, we inhibited SLC1A5 production using SLC1A5 inhibitor (GPNA) and determined the consequential effects on glutamine uptake, cell proliferation and cell apoptosis in both MDA-MB-231 and BT-549 cells. Results first showed the high expression of SLC1A5 in BC tissues and cells, as compared with matched healthy breast tissues and MCF-10A cells (Figure 3a-C). As presented in Figure 3d, glutamine consumption was inhibited after GPNA treatment. Comparatively, GPNA treatment induced proliferation repression of MDA-MB-231 and BT-549 cells (Figure 3e-G). On the opposite, the apoptosis of MDA-MB-231 and BT-549 cells was promoted after GPNA treatment (Figure 3h). In support, GPNA induced cell arrest at G0/G1 phase (Figure 3i), which also indicated the repressing role of SLC1A5 knockdown in cell proliferation. Furthermore, it was confirmed using Western blot analysis that GPNA reduced the protein expression of PCNA and Bcl-2, but increased Bax expression (Figure 3j). Besides, the above results were verified in both MDA-MB-231 and BT-549 cells transfected with the small hairpin RNA targeting SLC1A5 (sh-SLC1A5). The high efficiency of SLC1A5 knockdown was displayed in Supplementary Figure A. Clearly, SLC1A5 silencing inhibited glutamine uptake and cell proliferation, and induced cell apoptosis (Supplementary Figure B-G). The protein expression of PCNA and Bcl-2 was decreased, and Bax was increased after SLC1A5 knockdown (Supplementary Figure H). Collectively, SLC1A5 may act an oncogene in BC progression.

### CircSEPT9 regulated BC cell malignancy by interacting with SLC1A5

**Figure 4.**
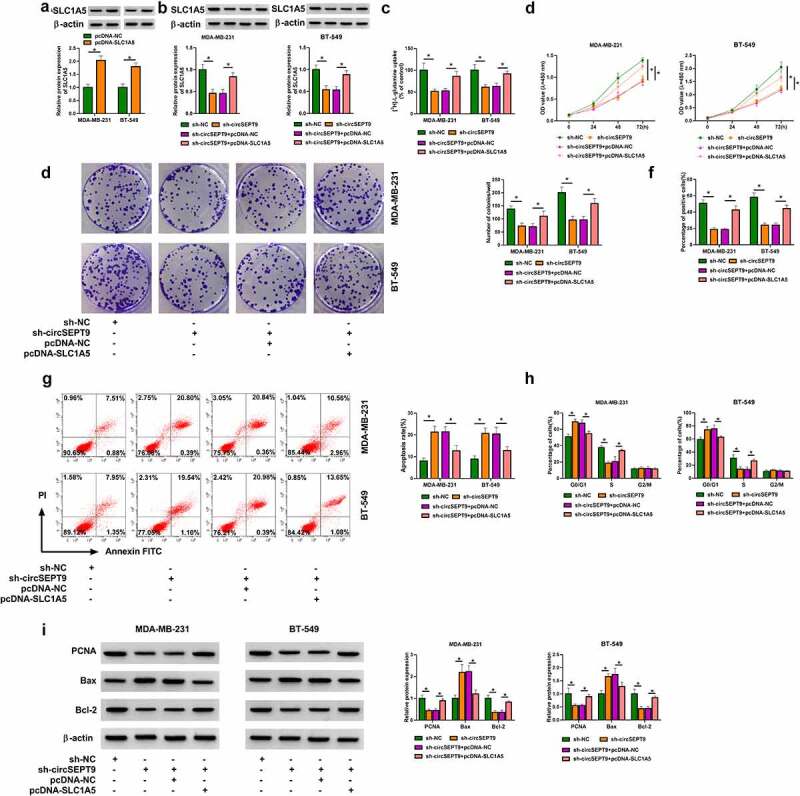
**The effects between circSEPT9 knockdown and SLC1A5 overexpression on BC cell malignancy**. (a) Western blot analysis was employed to determine the efficiency of SLC1A5 overexpression in MDA-MB-231 and BT-549 cells. (b-i) Both MDA-MB-231 and BT-549 cells were transfected with sh-NC, sh-circSEPT9, sh-circSEPT9+ pcDNA-NC and sh-circSEPT9+ pcDNA-SLC1A5, respectively, and SLC1A5 protein expression was determined by Western blot (b), glutamine uptake by glutamine uptake assay (c), cell proliferation by CCK-8, cell colony formation, EDU and DNA content quantitation assays (D, E, F and H), cell apoptosis by flow cytometry (g), and the protein expression of PCNA, Bax and Bcl-2 by Western blot (i). *P < 0.05

The study continued to analyze whether SLC1A5 participated in the regulation of circSEPT9 in the malignant progression of BC cells. The overexpression efficiency of SLC1A5 was detected by Western blot, and the results were presented in Figure 4a. Then, reduced expression of circSEPT9 inhibited SLC1A5 production, whereas the effect was attenuated after SLC1A5 overexpression (Figure 4b). The decreased glutamine uptake and cell proliferation caused by circSEPT9 depletion were relieved after increase of SLC1A5 expression (Figure 4c-F). As expected, circSEPT9 knockdown-induced apoptosis and cell cycle arrest (at G0/G1 phase) of MDA-MB-231 and BT-549 cells were remitted by increased expression of SLC1A5 (Figure 4g and H). Comparatively, enforced expression of SLC1A5 rescued circSEPT9 depletion-induced PCNA and Bcl-2 expression inhibition and Bax expression promotion (Figure 4i). By the large, these data manifest that SLC1A5 is associated with circSEPT9-mediated BC cell malignancy.

### CircSEPT9 regulated SLC1A5 expression by binding to miR-149-5p

**Figure 5.**
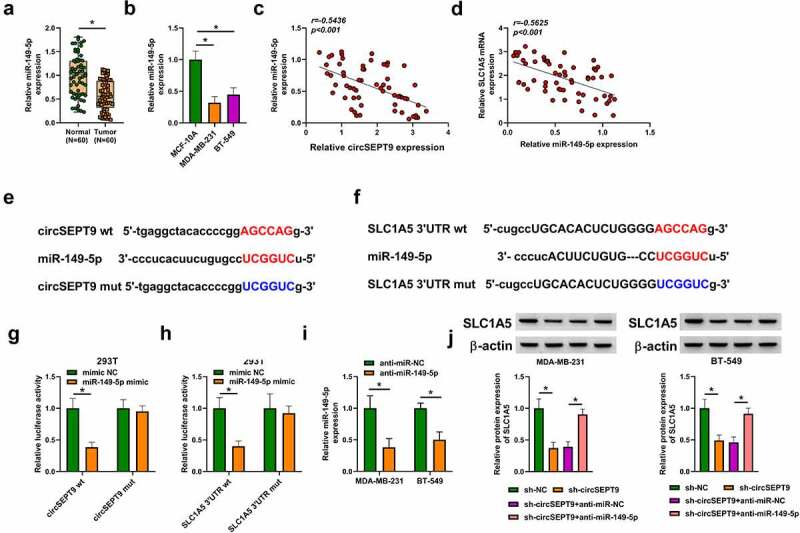
**CircSEPT9 regulated SLC1A5 expression through interaction with miR-149-5p**. (a and b) MiR-149-5p expression was determined by qRT-PCR in 60 pairs of BC tissues, paracancerous healthy breast tissues, MCF-10A cells, MDA-MB-231 cells and BT-549 cells. (c and d) The linear correlation of miR-149-5p and circSEPT9 or SLC1A5 was analyzed by Spearman correlation analysis in BC tissues. (e and f) The complementary sites of miR-149-5p with circSEPT9 and SLC1A5. (g and h) Dual-luciferase reporter assay was employed to determine the interaction of miR-149-5p with circSEPT9 and SLC1A5. (i) The efficiency of miR-149-5p knockdown was checked by qRT-PCR in MDA-MB-231 and BT-549 cells. (j) The effects between circSEPT9 knockdown and miR-149-5p depletion on SLC1A5 protein expression were revealed by Western blot. *P < 0.05

The miRNA interacted with both circSEPT9 and SLC1A5 was explored in this part. MiR-149-5p, a candidate, was found to be lowly expressed in BC tissues and cells (MDA-MB-231 and BT-549 cells), when compared with controls (Figure 5a and B). Additionally, the association between miR-149-5p and SLC1A5/circSEPT9 expression in BC tissues was determined by Spearman correlation analysis. As revealed in Figure 5c and D, miR-149-5p expression was negatively correlated with circSEPT9 and SLC1A5. Based on the above evidence, miR-149-5p was employed as a follow-up subject. The complementary sites of miR-149-5p with circSEPT9 and SLC1A5 were predicted by circbank and Starbase online databases, respectively, and presented in Figure 5e and F. Subsequently, the binding relationships were identified by a dual-luciferase reporter assay. As a result, we found that miR-149-5p introduction inhibited the luciferase activity of wild-type plasmids of circSEPT9 and SLC1A5, but not the luciferase activity of the mutants (circSEPT9 mut and SLC1A5 3ʹUTR mut) (Figure 5g and H), which suggests that circSEPT9 acts as a miR-149-5p sponge, and miR-149-5p targeted SLC1A5. Furthermore, we silenced both circSEPT9 and miR-149-5p to determine the consequential effects on SLC1A5 protein expression. The efficiency of miR-149-5p depletion was displayed in Figure 5i. As shown in Figure 5j, circSEPT9 knockdown reduced SLC1A5 expression, whereas the effect was remitted after decrease of miR-149-5p expression. Collectively, all data explain that circSEPT9 modulates SLC1A5 by interacting with miR-149-5p.

### *CircSEPT9 knockdown inhibited tumor tumorigenesis* in vivo

**Figure 6.**
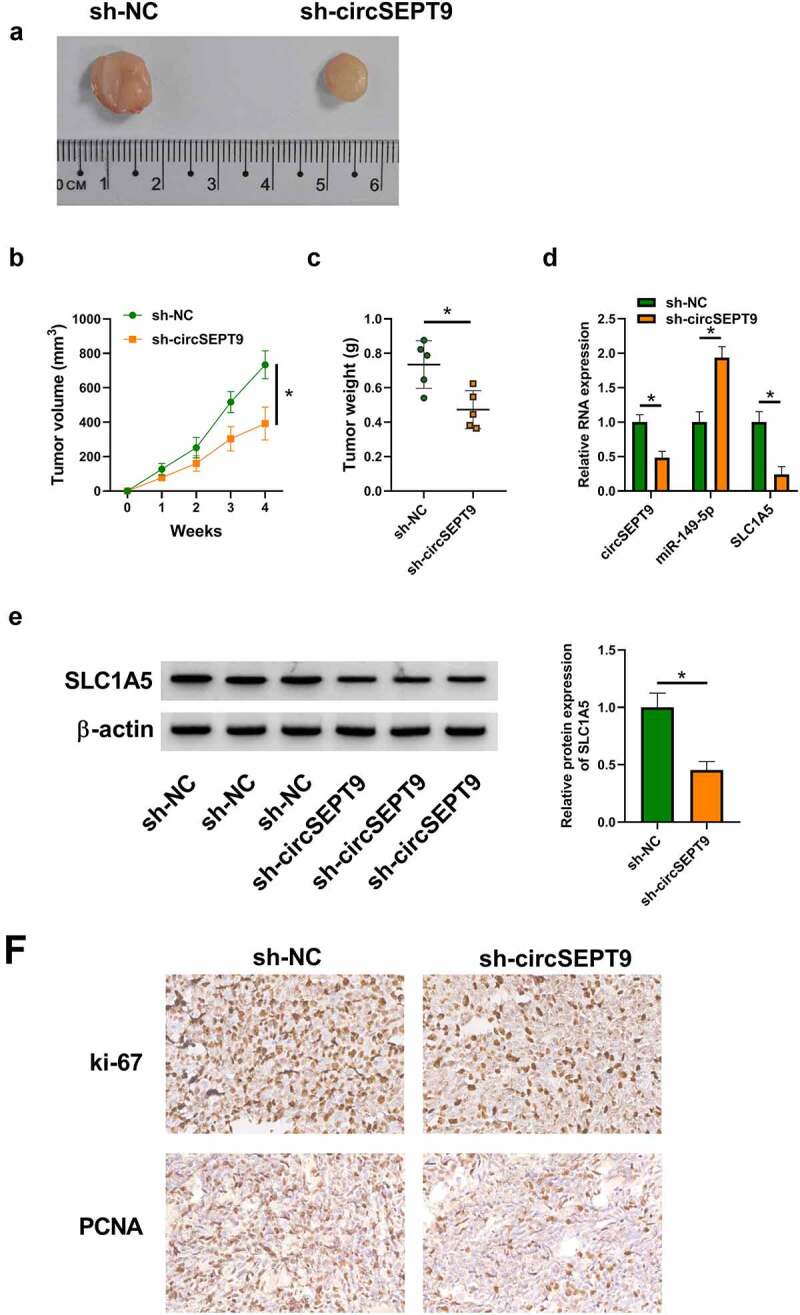
**The effect of circSEPT9 silencing on tumor formation *in vivo***. (a-c) The effect of circSEPT9 knockdown on tumor formation. (d and e) CircSEPT9, miR-149-5p and SLC1A5 expression were determined by qRT-PCR or Western blot analysis in the primary tumors from sh-circSEPT9 and sh-NC groups. (f) The effects of circSEPT9 depletion on the production of ki-67 and PCNA were analyzed by IHC assay in the primary tumors from MDA-MB-231 cells. *P < 0.05

The anti-cancer activities of circSEPT9 silencing *in vitro* were further testified by a mouse model assay. As shown in Figure 6a-C, circSEPT9 knockdown led to reduced tumor volume and weight. Comparatively, circSEPT9 depletion decreased the expression of circSEPT9 and SLC1A5, and increased miR-149-5p expression in the primary tumors from MDA-MB-231 cells (Figure 6d and E). Additionally, the production of proliferation-related markers, ki-67 and PCNA, was checked by IHC assay in the forming subcutaneous tumors. As expected, circSEPT9 depletion reduced the positive expression rates of the two proteins in the forming tumors from sh-circSEPT9 group, as compared with the tumors from sh-NC groups (figure 6f). In a word, these data demonstrates the repressing effect of circSEPT9 knockdown on tumor tumorigenesis *in vivo*.

## Discussion

Generally generated by head-to-tail splicing of mRNA, circRNA is a structurally stable transcript and its importance has been demonstrated in various types of cancers. Through high-throughput RNA sequencing, more circRNAs were discovered to be increased in BC, unlike the researches in GC that indicated more decreased circRNAs in expression than increased ones. In addition, some circRNAs have the potential as prognostic biomarkers for BC, such as circRNA epithelial stromal interaction 1 (circEPSTI1) [30], circRNA polo like kinase 1 (circPLK1) [31] and circRNA kinesin family member 4A (circKIF4A) [32]. In the present study, we explored the role of circSEPT9 in BC progression, and found that circSEPT9 might act as a promoter in BC malignant progression. Further analysis suggests that miR-149-5p and SLC1A5 are required for the regulation of circSEPT9 toward BC development.

More and more data reported that circRNA was clinically significant for the development of BC [33,34]. CircSEPT9, also named as circ_0005320, has been revealed to promote glioma cell malignancy by targeting miR-432-5p [35]. In the work conducted by Ju *et al*., we found that circSEPT9 was significantly upregulated in metastatic oral mucosal melanoma (OMM) tissues, and might function as a competing endogenous RNA to mediate metastatic property of OMM [36]. Besides, circSEPT9 silencing inhibited BC cell motility and promoted autophagy through binding to miR-637 [37]. Herein, we analyzed association between clinicopathological variables and circSEPT9 expression in BC patients, and found that circSEPT9 expression was significantly associated with tumor size, distant metastasis, TNM stage and HER-2 status. We reported the high expression of circSEPT9 in BC cells. CircSEPT9 knockdown led to inhibition of BC cell proliferation, formation of primary tumors from BC cells, and promotion of cell apoptosis, which was supported by the published data [37]. Besides, the study demonstrates that circSEPT9 depletion represses the malignant behaviors of BC cells by reducing glutamine uptake.

Some mechanisms regarding the regulation of SLC1A5 in BC progression have been well revealed. For example, SLC1A5 inhibitor repressed glutamine consumption, resulting in the inhibition of BC cell proliferation [16]. Glutamate dehydrogenase (GLUD1), an important enzyme in glutaminolysis, is associated with BC molecular subtypes [38], and recent study suggests that SLC1A5 may regulate GLUD1 expression in BC [39]. Besides, it was found that SLC1A5 was involved in the endocrine therapy failure in luminal BC, co-expressed with transaldolase 1 [40]. In this work, we found that the downregulation of SLC1A5 expression inhibited glutamine uptake, cell proliferation and induced cell apoptosis in BC cells *in vitro* using the inhibitor of SLC1A5, GPNA, and the small hairpin RNA targeting SLC1A5. Importantly, the study revealed that SLC1A5 was negatively regulated by circSEPT9, and its introduction attenuated the effects of circSEPT9 silencing on BC cell malignancy, which indicates the association both circSEPT9 and SLC1A5 in BC cell malignancy.

A well-documented mechanism of circRNAs in regulating cancer progression involves its function as miRNA sponges, further protecting target mRNA. To analyze the mechanism responsible for the effects of both circSEPT9 and SLC1A5 on BC progression, we screened the miRNA able to bind to the two regulators. MiR-149-5p is a small miRNA that has been indicated as a tumor repressor in many malignancies [41,42]. With respect to the mechanism of BC progression, circ_0072995 contributed to BC tumorigenesis by directing binding to miR-149-5p [43]. MiR-149-5p could be upregulated under propofol, and its depletion relieved propofol-induced effects in BC cells [44]. The above evidence supported miR-149-5p as a candidate of subsequent study. As predicted by online databases and identified by a dual-luciferase reporter assay, miR-149-5p bound to circSEPT9 and SLC1A5. Meanwhile, Western blot analysis displayed that circSEPT9 knockdown reduced SLC1A5 expression, which was attenuated after miR-149-5p depletion, suggesting that circSEPT9 regulates SLC1A5 through miR-149-5p.

The above results demonstrate that circSEPT9 regulates BC cell malignancy through SLC1A5/miR-149-5p pathway. Nevertheless, the present study lacks the data about the effects between miR-149-5p and circSEPT9/SLC1A5 on BC development *in vitro*. Besides, there is no direct evidence regarding the mechanism of SLC1A5 in regulating BC progression in the present study. We will design function experiments to address the above two issues in future.

## Conclusion

Our data suggest that circSEPT9 may act as an oncogenic role in BC progression. The underlying mechanism is that circSEPT9 sponges miR-149-5p to stimulate SLC1A5 production, so as to increase glutamine uptake, thereby promoting proliferation and inhibiting apoptosis of BC cells. The novel findings make circSEPT9 an attractive candidate for the therapy of BC.

## Supplementary Material

Supplemental Material

## References

[1] DeSantis CE, Ma J, Goding Sauer A, et al. Breast cancer statistics, 2017, racial disparity in mortality by state. CA Cancer J Clin. 2017;67(6):439–448. 28972651 10.3322/caac.21412

[2] Bray F, Ferlay J, Soerjomataram I, et al. Global cancer statistics 2018: GLOBOCAN estimates of incidence and mortality worldwide for 36 cancers in 185 countries. CA Cancer J Clin. 2018;68(6):394–424. 30207593 10.3322/caac.21492

[3] van Diest PJ, Van Der Wall E, Baak JP. Prognostic value of proliferation in invasive breast cancer: a review. J Clin Pathol. 2004;57(7):675–681. 15220356 10.1136/jcp.2003.010777 PMC1770351

[4] Okholm TLH, Nielsen MM, Hamilton MP, et al. Circular RNA expression is abundant and correlated to aggressiveness in early-stage bladder cancer. NPJ Genom Med. 2017;2:36. 29263845 10.1038/s41525-017-0038-z PMC5705701

[5] Kristensen LS, Andersen MS, Stagsted LVW, et al. The biogenesis, biology and characterization of circular RNAs. Nat Rev Genet. 2019;20(11):675–691. 31395983 10.1038/s41576-019-0158-7

[6] Wang Y, Mo Y, Gong Z, *et al*. Circular RNAs in human cancer. Mol Cancer. 2017;16(1):25. 28143578 10.1186/s12943-017-0598-7 PMC5282898

[7] Liu Z, Zhou Y, Liang G, et al. Circular RNA hsa_circ_001783 regulates breast cancer progression via sponging miR-200c-3p. Cell Death Dis. 2019;10(2):55. 30670688 10.1038/s41419-018-1287-1 PMC6343010

[8] Zhang J, Ke S, Zheng W, et al. Hsa_circ_0003645 promotes breast cancer progression by regulating miR-139-3p/HMGB1 axis. Onco Targets Ther. 2020;13:10361–10372. 33116616 10.2147/OTT.S265796 PMC7568624

[9] Qiu X, Wang Q, Song H, et al. circ_103809 promotes breast cancer progression by regulating the PI3K/AKT signaling pathway. Oncol Lett. 2020;19(6):3725–3730. 32382325 10.3892/ol.2020.11507 PMC7202270

[10] Butler EB, Zhao Y, Muñoz-Pinedo C, et al. Stalling the engine of resistance: targeting cancer metabolism to overcome therapeutic resistance. Cancer Res. 2013;73(9):2709–2717. 23610447 10.1158/0008-5472.CAN-12-3009 PMC3644012

[11] Hensley CT, Wasti AT, DeBerardinis RJ. Glutamine and cancer: cell biology, physiology, and clinical opportunities. J Clin Invest. 2013;123(9):3678–3684. 23999442 10.1172/JCI69600 PMC3754270

[12] Kaadige MR, Looper RE, Kamalanaadhan S, et al. Glutamine-dependent anapleurosis dictates glucose uptake and cell growth by regulating MondoA transcriptional activity. Proc Natl Acad Sci U S A. 2009;106(35):14878–14883. 19706488 10.1073/pnas.0901221106 PMC2736411

[13] Yuneva MO, Fan TW, Allen TD, *et al*. The metabolic profile of tumors depends on both the responsible genetic lesion and tissue type. Cell Metab. 2012;15(2):157–170. 22326218 10.1016/j.cmet.2011.12.015 PMC3282107

[14] Kanai Y, Hediger MA. The glutamate/neutral amino acid transporter family SLC1: molecular, physiological and pharmacological aspects. Pflugers Arch. 2004;447(5):469–479. 14530974 10.1007/s00424-003-1146-4

[15] Huang F, Zhao Y, Zhao J, et al. Upregulated SLC1A5 promotes cell growth and survival in colorectal cancer. Int J Clin Exp Pathol. 2014;7(9):6006–6014. 25337245 PMC4203216

[16] van Geldermalsen M, Wang Q, Nagarajah R, *et al*. ASCT2/SLC1A5 controls glutamine uptake and tumour growth in triple-negative basal-like breast cancer. Oncogene. 2016;35(24):3201–3208. 26455325 10.1038/onc.2015.381 PMC4914826

[17] Friedman RC, Farh KK, Burge CB, et al. Most mammalian mRNAs are conserved targets of microRNAs. Genome Res. 2009;19(1):92–105. 18955434 10.1101/gr.082701.108 PMC2612969

[18] Chen J, Jiang Q, Jiang XQ, et al. miR-146a promoted breast cancer proliferation and invasion by regulating NM23-H1. J Biochem. 2020;167(1):41–48. 31598678 10.1093/jb/mvz079

[19] Ruan L, Qian X. MiR-16-5p inhibits breast cancer by reducing AKT3 to restrain NF-κB pathway. Biosci Rep. 2019;39(8):8. 10.1042/BSR20191611 PMC6706597 31383783

[20] Hui C, Tian L, He X. Circular RNA circNHSL1 Contributes to Gastric Cancer Progression Through the miR-149-5p/YWHAZ Axis. Cancer Manag Res. 2020;12:7117–7130. 32848466 10.2147/CMAR.S253152 PMC7429192

[21] Meng X, Sun W, Yu J, et al. LINC00460-miR-149-5p/miR-150-5p-mutant p53 feedback loop promotes oxaliplatin resistance in colorectal cancer. Mol Ther Nucleic Acids. 2020;22:1004–1015. 33251049 10.1016/j.omtn.2020.10.018 PMC7679243

[22] Xu M, Xiao J, Chen M, et al. miR‑149‑5p promotes chemotherapeutic resistance in ovarian cancer via the inactivation of the Hippo signaling pathway. Int J Oncol. 2018;52(3):815–827. 29393390 10.3892/ijo.2018.4252 PMC5807033

[23] Sánchez-González I, Bobien A, Molnar C, et al. miR-149 suppresses breast cancer metastasis by blocking paracrine interactions with macrophages. Cancer Res. 2020;80(6):1330–1341. 31911555 10.1158/0008-5472.CAN-19-1934

[24] Lu H, Li X, Lu Y, et al. ASCT2 (SLC1A5) is an EGFR-associated protein that can be co-targeted by cetuximab to sensitize cancer cells to ROS-induced apoptosis. Cancer Lett. 2016;381(1):23–30. 27450723 10.1016/j.canlet.2016.07.020 PMC5017913

[25] Wu Y, Xie Z, Chen J, *et al*. Circular RNA circTADA2A promotes osteosarcoma progression and metastasis by sponging miR-203a-3p and regulating CREB3 expression. Mol Cancer. 2019;18(1):73. 30940151 10.1186/s12943-019-1007-1 PMC6444890

[26] Pan G, Mao A, Liu J, et al. Circular RNA hsa_circ_0061825 (circ-TFF1) contributes to breast cancer progression through targeting miR-326/TFF1 signalling. Cell Prolif. 2020;53(2). e12720-e12720. doi:10.1111/cpr.12720. PMC7048212 31961997

[27] Lu H, Li X, Luo Z, et al. Cetuximab reverses the Warburg effect by inhibiting HIF-1-regulated LDH-A. Mol Cancer Ther. 2013;12(10):2187–2199. 23920275 10.1158/1535-7163.MCT-12-1245 PMC3811007

[28] Qiu N, Xu X, He Y. LncRNA TUG1 alleviates sepsis-induced acute lung injury by targeting miR-34b-5p/GAB1. BMC Pulm Med. 2020;20(1):49. 32087725 10.1186/s12890-020-1084-3 PMC7036216

[29] Huang D-W, Huang M, Lin X-S, et al. CD155 expression and its correlation with clinicopathologic characteristics, angiogenesis, and prognosis in human cholangiocarcinoma. Onco Targets Ther. 2017;10:3817–3825. 28814880 10.2147/OTT.S141476 PMC5546808

[30] Chen B, Wei W, Huang X, et al. circEPSTI1 as a prognostic marker and mediator of triple-negative breast cancer progression. Theranostics. 2018;8(14):4003–4015. 30083277 10.7150/thno.24106 PMC6071524

[31] Kong Y, Yang L, Wei W, et al. CircPLK1 sponges miR-296-5p to facilitate triple-negative breast cancer progression. Epigenomics. 2019;11(10):1163–1176. 31337246 10.2217/epi-2019-0093

[32] Tang H, Huang X, Wang J, et al. circKIF4A acts as a prognostic factor and mediator to regulate the progression of triple-negative breast cancer. Mol Cancer. 2019;18(1):23. 30744636 10.1186/s12943-019-0946-x PMC6369546

[33] Liang G, Ling Y, Mehrpour M, et al. Autophagy-associated circRNA circCDYL augments autophagy and promotes breast cancer progression. Mol Cancer. 2020;19(1):65. 32213200 10.1186/s12943-020-01152-2 PMC7093993

[34] Song H, Sun J, Kong W, et al. Construction of a circRNA-related ceRNA prognostic regulatory network in breast cancer. Onco Targets Ther. 2020;13:8347–8358. 32922032 10.2147/OTT.S266507 PMC7455596

[35] Yue L, Wang G, Zhu M. CircRNA SEPT9 contributes to malignant behaviors of glioma cells via miR-432-5p-mediated regulation of LASP1. Brain Res. 2021;1766:147501. 33915163 10.1016/j.brainres.2021.147501

[36] Ju H, Zhang L, Mao L, et al. Altered expression pattern of circular RNAs in metastatic oral mucosal melanoma. Am J Cancer Res. 2018;8(9):1788–1800. 30323971 PMC6176177

[37] Zheng X, Huang M, Xing L, et al. The circRNA circSEPT9 mediated by E2F1 and EIF4A3 facilitates the carcinogenesis and development of triple-negative breast cancer. Mol Cancer. 2020;19(1):73. 32264877 10.1186/s12943-020-01183-9 PMC7137343

[38] Hou H, Luo C, Chen Z, et al. [Correlation of glutamate dehydrogenase with several tumors]. Sheng Wu Gong Cheng Xue Bao. 2019;35(3):389–395. 30912347 10.13345/j.cjb.180281

[39] Craze ML, El-Ansari R, Aleskandarany MA, *et al*. Glutamate dehydrogenase (GLUD1) expression in breast cancer. Breast Cancer Res Treat. 2019;174(1):79–91. 30470977 10.1007/s10549-018-5060-z

[40] Alfarsi LH, El Ansari R, Craze ML, et al. SLC1A5 co-expression with TALDO1 associates with endocrine therapy failure in estrogen receptor-positive breast cancer. Breast Cancer Res Treat. 2021;189(2):317–331. 34282517 10.1007/s10549-021-06298-1 PMC8357718

[41] Kong YG, Cui M, Chen SM, et al. LncRNA-LINC00460 facilitates nasopharyngeal carcinoma tumorigenesis through sponging miR-149-5p to up-regulate IL6. Gene. 2018;639:77–84. 28987345 10.1016/j.gene.2017.10.006

[42] Ye X, Chen X. miR-149-5p inhibits cell proliferation and invasion through targeting GIT1 in medullary thyroid carcinoma. Oncol Lett. 2019;17(1):372–378. 30655777 10.3892/ol.2018.9628 PMC6313157

[43] Qi C, Qin X, Zhou Z, et al. Circ_0072995 promotes cell carcinogenesis via up-regulating miR-149-5p-mediated SHMT2 in breast cancer. Cancer Manag Res. 2020;12:11169–11181. 33173349 10.2147/CMAR.S272274 PMC7648565

[44] Tian D, Tian M, Ma ZM, et al. Anesthetic propofol epigenetically regulates breast cancer trastuzumab resistance through IL-6/miR-149-5p axis. Sci Rep. 2020;10(1):8858. 32483313 10.1038/s41598-020-65649-y PMC7264192

