# Supplementary material for: Knockdown of circular RNA septin 9 inhibits the malignant progression of breast cancer by reducing the expression of solute carrier family 1 member 5 in a microRNA-149-5p-dependent manner
Source: Bioengineered. 2021 Dec 11;12(2):10624–37. doi: 10.1080/21655979.2021.2000731 (PMC8809977; doi:10.1080/21655979.2021.2000731)
Supplement: Supplemental Material [file KBIE_A_2000731_SM6932.zip › supplementary/Supplementary_fig_revised legend.docx]

**Supplementary Figure The effects of SLC1A5 knockdown on BC cell processes.** (A-H) Both MDA-MB-231 and BT-549 cells were transfected with sh-con and sh-SLC1A5, respectively, and SLC1A5 protein expression was determined by Western blot (A), glutamine uptake by glutamine uptake assay (B), cell proliferation by CCK-8, cell colony formation, EDU and DNA content quantitation assays (C, D, E and G), cell apoptosis by flow cytometry (F), and the protein expression of PCNA, Bax and Bcl-2 by Western blot (H). **P*<0.05.
