# Supplementary figures and images for: Knockdown of circular RNA septin 9 inhibits the malignant progression of breast cancer by reducing the expression of solute carrier family 1 member 5 in a microRNA-149-5p-dependent manner
Source: Bioengineered. 2021 Dec 11;12(2):10624–37. doi: 10.1080/21655979.2021.2000731 (PMC8809977; doi:10.1080/21655979.2021.2000731)

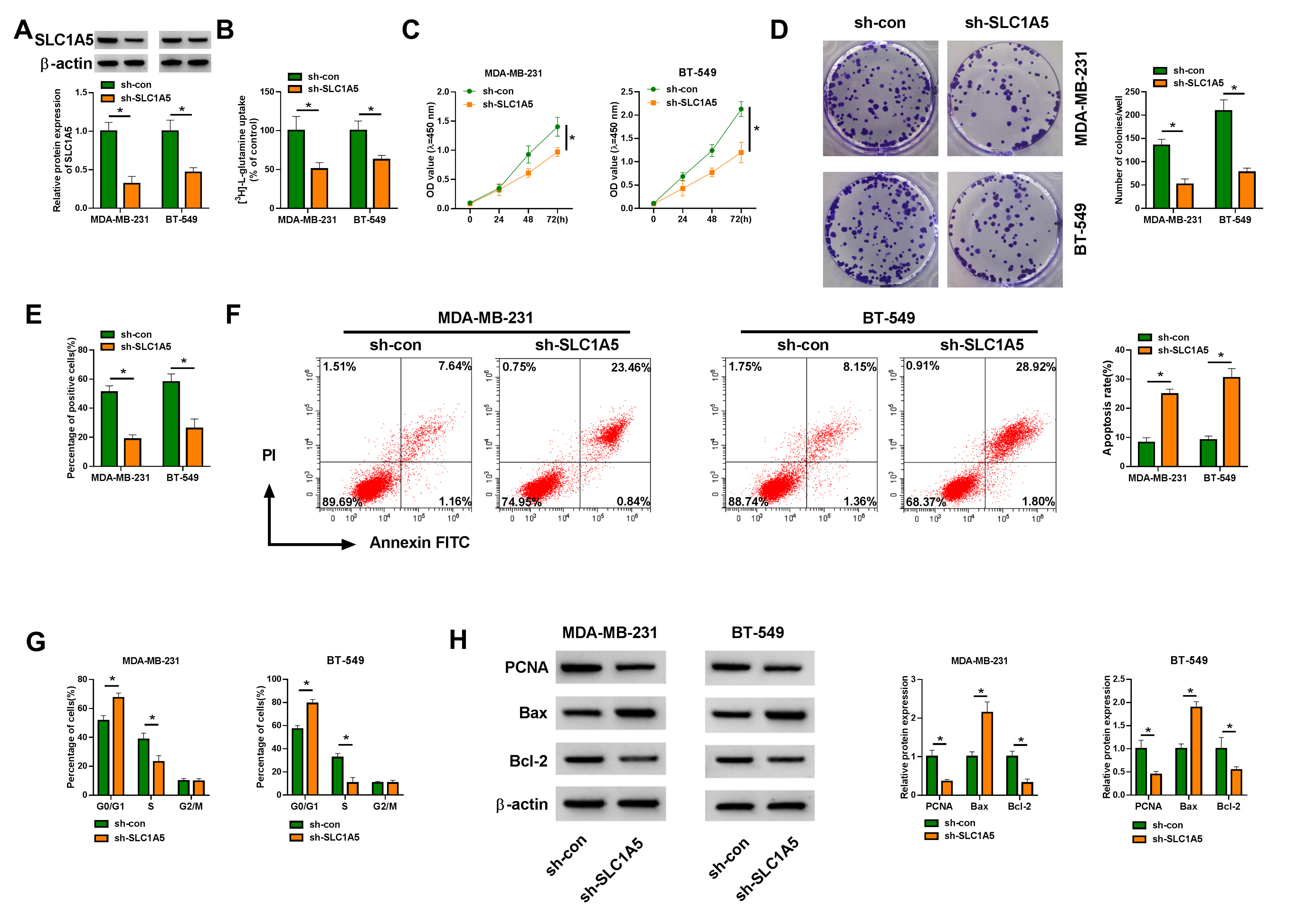

Supplement: Supplemental Material [file KBIE_A_2000731_SM6932.zip › supplementary/Supplementary_fig_revised.tif]
